# Curcumin mediated suppression of nuclear factor-κB promotes chondrogenic differentiation of mesenchymal stem cells in a high-density co-culture microenvironment

**DOI:** 10.1186/ar3065

**Published:** 2010-07-01

**Authors:** Constanze Buhrmann, Ali Mobasheri, Ulrike Matis, Mehdi Shakibaei

**Affiliations:** 1 Musculoskeletal Research Group, Institute of Anatomy, Ludwig-Maximilians-University Munich, Pettenkoferstrasse 11, D-80336 Munich, Germany; 2 Division of Veterinary Medicine, School of Veterinary Medicine and Science, Faculty of Medicine and Health Sciences, University of Nottingham, Sutton Bonington Campus, Sutton Bonington LE12 5RD, UK; 3 Clinic of Veterinary Surgery, Ludwig-Maximilians-University Munich, Veterinärstr. 13, 80539 Munich, Germany

## Abstract

**Introduction:**

Osteoarthritis (OA) and rheumatoid arthritis (RA) are characterised by joint inflammation and cartilage degradation. Although mesenchymal stem cell (MSC)-like progenitors are resident in the superficial zone of articular cartilage, damaged tissue does not possess the capacity for regeneration. The high levels of pro-inflammatory cytokines present in OA/RA joints may impede the chondrogenic differentiation of these progenitors. Interleukin (IL)-1β activates the transcription factor nuclear factor-κB (NF-κB), which in turn activates proteins involved in matrix degradation, inflammation and apoptosis. Curcumin is a phytochemical capable of inhibiting IL-1β-induced activation of NF-κB and expression of apoptotic and pro-inflammatory genes in chondrocytes. Therefore, the aim of the present study was to evaluate the influence of curcumin on IL-1β-induced NF-κB signalling pathway in MSCs during chondrogenic differentiation.

**Methods:**

MSCs were either cultured in a ratio of 1:1 with primary chondrocytes in high-density culture or cultured alone in monolayer with/without curcumin and/or IL-1β.

**Results:**

We demonstrate that although curcumin alone does not have chondrogenic effects on MSCs, it inhibits IL-1β-induced activation of NF-κB, activation of caspase-3 and cyclooxygenase-2 in MSCs time and concentration dependently, as it does in chondrocytes. In IL-1β stimulated co-cultures, four-hour pre-treatment with curcumin significantly enhanced the production of collagen type II, cartilage specific proteoglycans (CSPGs), β1-integrin, as well as activating MAPKinase signaling and suppressing caspase-3 and cyclooxygenase-2.

**Conclusions:**

Curcumin treatment may help establish a microenvironment in which the effects of pro-inflammatory cytokines are antagonized, thus facilitating chondrogenesis of MSC-like progenitor cells *in vivo*. This strategy may support the regeneration of articular cartilage.

## Introduction

Osteoarthritis (OA) and rheumatoid arthritis (RA) involve degenerative changes in the joint, leading to loss of function, pain and significant disability [1]. OA and RA are not only common joint diseases in the elderly population but increasingly they affect young individuals. Collectively, they represent a large proportion of orthopaedic cases [2]. Articular cartilage is an avascular, alymphatic and aneural tissue with bradytrophic characteristics and a very poor capacity for self-repair and regeneration [3]. Cartilage repair is ineffective and often leads to replacement of the articular cartilage by a mechanically inferior fibrocartilage tissue thus promoting progressive degeneration and impairment of joint function [4]. This inherent weakness in cartilage repair highlights the acute need for novel treatments using tissue engineering and regenerative medicine, and innovative new regenerative strategies that involve stimulation of articular cartilage repair *in vivo*.

OA is characterized by an imbalance between cartilage anabolism and catabolism. The local production and release of pro-inflammatory cytokines (interleukin-1β (IL-1β), interleukin-6 (IL-6), tumor necrosis factor-α (TNF-α)) play a central role in the pathogenesis of OA [5-7]. It is well known that IL-1β and TNF-α production activates the transcription factor nuclear factor-κB (NF-κB) in chondrocytes. Once activated, NF-κB translocates into the nucleus, where it induces the expression of distinct subsets of genes encoding inflammatory, apoptotic and extracellular matrix (ECM) degrading enzymes. NF-κB activates the expression of matrix degrading enzymes such as matrix metalloproteinases (MMPs) and enzymes responsible for production of prostaglandins (that is, cyclooxygenase-2 (COX-2)) leading to enhanced degradation of the ECM and induction of pain [8]. Additionally, in articular chondrocytes, NF-κB stimulates the production of pro-inflammatory catabolic cytokines, which induce apoptosis through activation of the pro-apoptotic enzyme caspase-3 and cleavage of the DNA repair enzyme poly(ADP-ribose)polymerase (PARP) [9].

During embryonic development, cartilage develops from mesenchymal stem cells (MSCs) by condensation and differentiation. Recent studies have shown that MSC-like progenitors also exist in the superficial zone of articular cartilage and that their abundance in arthritic cartilage is elevated [10]. Despite this, cartilage regeneration *in vivo *is inefficient and the resulting fibrocartilage is structurally and functionally inadequate. A possible explanation for this lack of regeneration is that the ongoing inflammatory processes that occur during the course of OA/RA result in higher synovial and circulating levels of pro-inflammatory cytokines, which may in turn impede the chondrogenic differentiation of cartilage resident progenitors. Therefore, blocking the pro-inflammatory cytokine induced cartilage degeneration and inflammatory cascades might create a more suitable microenvironment for the chondrogenesis of MSC-like progenitors.

In recent years the phytochemical curcumin has been identified as a potent anti-inflammatory substance in several diseases such as cancer, inflammatory bowel disease, pancreatitis, chronic anterior uveitis and arthritis [9,11-16]. Curcumin is a natural yellow orange dye derived from the rhizome of *Curcuma longa*. It is insoluble in water but is soluble in ethanol, dimethylsulfoxide and other organic solvents. It has a melting point of 183°C and a molecular weight of 368.37. Commercial curcumin contains three major components: Diferuloylmethane (82%) and its derivatives demethoxycurcumin (15%) and bisdemethoxycurcumin (3%), together referred to as curcuminoids [9,11-16], all of which have anti-inflammatory activity. Curcumin reduces tumor cell survival, tumor expansion and secondary inflammation via NF-κB inhibition [13,17]. Further, it suppresses constitutive IκBα phosphorylation through the inhibition of IκB kinase [13,18]. There is increasing interest in curcumin as a therapeutic option for OA and RA, with evidence that curcumin inhibits the IL-1β-induced activation of NF-κB in human articular chondrocytes [9,14]. Furthermore, in a recent study we have demonstrated that curcumin exerts anti-apoptotic effects on IL-1β stimulated human chondrocytes through inhibition of caspase-3 activation and PARP cleavage [15].

The aim of the present investigation was to evaluate whether IL-1β stimulated MSCs (either alone or in a co-culture model of OA with primary chondrocytes) pre-treated with curcumin may impede the adverse effects of this pro-inflammatory cytokine and create a more suitable microenvironment for the chondrogenic differentiation of cartilage resident progenitor cells.

## Materials and methods

### Antibodies and reagents

Polyclonal anti-collagen type II antibody (PAB746), monoclonal anti-adult cartilage-specific proteoglycan antibody (MAB2015), anti-β1-integrin antibody (MAB1965), and alkaline phosphatase linked sheep anti-mouse (AP303A) and sheep anti-rabbit secondary antibodies (AP304A) for immunoblotting and immuno-electron labelling were purchased from Chemicon International, Inc. (Temecula, CA, USA). Monoclonal anti-β-Actin (A4700) was purchased from Sigma, St. Louis, MO, USA). Monoclonal anti-Sox-9 was purchased from Acris Antibodies GmbH, Hiddenhausen, Germany. Monoclonal anti-phospho-p42/p44 ERK1/2 antibody (610032) and polyclonal anti-Shc antibody (610082) were purchased from BD (BD Biosciences, Erembodegem, Belgium). Polyclonal anti-active caspase-3 (AF835) was purchased from R&D Systems (Abingdon, UK). Antibodies against phospho-specific IκBα (Ser 32/36) and against anti-phospho-specific p65(NF-κB)/(Ser536) were obtained from Cell Technology (Beverly, MA, USA). Curcumin with a purity > 95% was purchased from Indsaff (Punjab, India). This commercial source of curcumin contains three major components: Diferuloylmethane (the most abundant and active component of turmeric) (82%) and its derivatives demethoxycurcumin (15%) and bisdemethoxycurcumin (3%), together referred to as curcuminoids [9,11-16]. Curcumin was dissolved in dimethylsulfoxide (DMSO) as a stock concentration of 500 μM and stored at -80°C. Serial dilutions were prepared in culture medium.

### Cell culture

Mesenchymal stem cells (MSCs) were isolated from canine adipose tissue biopsies and primary canine chondrocytes were isolated from cartilage from the femoral head. Samples were obtained during total hip replacement surgery with fully-informed owner consent and ethical project approval from the ethical review committee of the Ludwig-Maximilians-University, Munich, Germany. Chondrocytes and MSCs used in co-culture experiments were always from the same animal. In total, the experiments were performed three times and samples from three different donors were used. Donor ages ranged from five to seven years.

Briefly, for MSCs isolation, adipose tissue was cut into small pieces and digested with collagenase 0.2% in Ham's-F12 in a water bath at 37°C for two hours. Digested adipose tissue was centrifuged at 1,000 g for five minutes and the pellet was resuspended in cell culture medium consisting of DMEM/Ham's-F12 1:1, 10% FCS, 1% partricin solution, 1% penicillin/streptomycin solution (10 000 IU/10 000 IU), 75 μg/ml ascorbic acid, 1% essential amino acids and 1% Glutamine, all obtained from Seromed (Munich, Germany) in a T75 cell culture flask and incubated at 37°C/5%CO_2_, 95% humidity. After four days, non-adherent cells were discarded by washing with Hank's salt solution. The medium was changed three times per week. Adherent cells were split following formation of fibroblast-like cell colonies and upon reaching 60 to 70% confluence, and were sub-cultured until the third or fourth passage was achieved. As there are no definitive MSC specific cellular markers, we identified them by their ability to adhere to tissue culture plastic *in vitro*, through their multilineage differentiation potential *in vitro *and through a combination of expression and lack of defined markers (CD105^+^, CD90^+^, CD45^-^, CD34^-^) [19-22].

For chondrocyte isolation the cartilage sample was sliced into 1 to 2 mm thick slices and incubated first with pronase (2%/Hams-F12) (Roche Diagnostics, Mannheim, Germany), followed by collagenase incubation (0.2%/Ham's-F12) (Sigma) in a shaking water bath at 37°C. The digested sample was centrifuged at 1,000 g for five minutes and cells plated at 1 × 10^6 ^cells per T75 flask at 37°C/5%CO_2_. The first medium change was performed after 24 hours, and the following medium changes three times per week. Chondrocytes were split at approximately 70% confluency and passaged twice.

### High-density culture

Three-dimensional high-density cultures at the air-liquid interface were prepared as previously described [23]. Cells were centrifuged at 1,000 g for five minutes and around one million cells (approximately 8 μl) from the cell pellet were pipetted directly onto a nitrocellulose filter on a steel grid. This model allows the cells to aggregate, forming a distinct pellet, which was examined after 14 days.

High-density culture pellets either consisted only of MSCs or primary chondrocytes, or a mixture of MSCs and primary chondrocytes (1:1) (co-culture). In all experiments, cultures and co-cultures were either incubated with cell culture medium (10% FCS) or with a chondrogenic induction medium as described by Pittenger *et al*. [24] consisting of DMEM base medium, D-(+)-glucose 0.35 g/100 ml (Sigma, Cat No. G7021), ITS+ 1 liquid media supplement (10 μg/ml insulin, 5.5 μg/ml transferrin, 5 ng/ml selenium, 0.5 mg/ml bovine albumin, 4.7 μg/ml linoleic acid (Sigma, Cat. No. I-2521), 0.1 mM ascorbate-2-phosphate (Sigma, Cat. No. A-8960), 10^-7 ^M dexamethasone (Sigma, Cat. No. D-8893), penicillin/streptomycin solution (10,000 IU/10,000 IU/100 ml). 10 ng/ml hTGFβ1 (Acris Antibodies GmbH, Hiddenhausen, Germany) were added fresh to the medium before each medium change. Furthermore, some cultures and co-cultures were then incubated with one of the following treatments: curcumin only; pre-stimulated with curcumin for four hours in suspension and then transferred to high-density culture; 10 ng/ml IL-1β; curcumin and IL-1β; pre-stimulated with curcumin for four hours in suspension and then brought into high-density culture and stimulated with IL-1β; or pre-stimulated with curcumin for four hours in suspension and then brought into high-density culture and stimulated with IL-1β and curcumin. Medium changes were made every three days.

### Time and concentration dependent experiments in monolayer culture

To examine in more detail the interaction between curcumin and IL-1β in MSCs and the pathological pathways involved, monolayer cultures of MSCs were evaluated. First, MSCs were cultured with various concentrations of curcumin (0, 0.5, 1, 2 and 5 μM) for four hours, followed by 24 hours 10 ng/ml IL-1β stimulation. Second, MSC cultures were pre-treated for four hours with 5 μM curcumin followed by one hour 10 ng/ml IL-1β stimulation. Whole cell lysates, cytoplasmic extracts and nuclear extracts were taken at various time points and evaluated with Western blotting.

### Electron microscopy

Transmission electron microscopy was performed as previously described [25]. High-density co-cultures were fixed for one hour in Karnovsky-fixative fixative and post-fixed in 1% OsO_4 _solution. After dehydration, pellets were embedded in Epon, ultrathin cuts were made on a Reichert-Ultracut E and contrasted with a mixture of 2% uranyl acetate/lead citrate. A transmission electron microscope (TEM 10, Zeiss, Jena, Germany) was used to examine the co-cultures. To quantify apoptosis, the number of cells exhibiting typical morphological features of apoptotic cell death was determined by scoring 100 cells from 30 different microscopic fields per culture and the number of apoptotic cells expressed as an indicator of MSC culture degradation.

### Western blot analysis

For Western blotting, total cell proteins were either extracted from the cell cultures with lysis buffer on ice for 30 minutes or nuclear extracts and cytoplasmic extracts prepared as previously described [9]. Total protein content was measured with the bicinchoninic acid system (Uptima, France) using bovine serum albumin as a standard. Samples were further reduced with 2-mercaptoethanol and total protein concentrations adjusted. Proteins (500 ng per lane total protein) were separated with SDS-PAGE under reducing conditions on 5%, 7%, 10% or 12% gels and then blotted onto nitrocellulose membranes using a trans blot apparatus (Bio-Rad, Munich, Germany). After blocking for two hours in 5% (w/v) skimmed milk powder in phosphate buffered saline (PBS)/0.1% Tween 20, membranes were incubated with the primary antibodies (overnight, 4°C), followed by incubation with the alkaline phosphatase conjugated secondary antibodies for two hours at room temperature. Finally, specific antigen-antibody complexes were detected using nitroblue tetrazolium and 5-bromo-4-chloro-3-indoylphosphate (*p*-toluidine salt; Pierce, Rockford, IL, USA) as substrates for alkaline phosphatase. Specific binding was quantified by densitometry using "Quantity One" (Bio-Rad Laboratories Inc. Munich, Germany).

## Results

### Characterisation of canine adipose tissue derived mesenchymal stem cells

**Figure 1:**
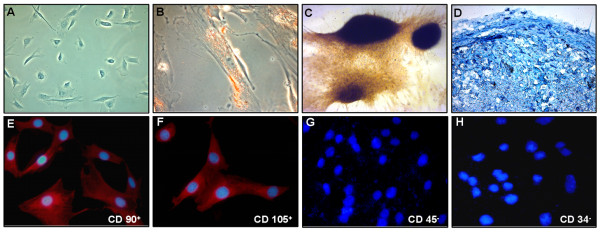
**Characterisation of MSCs**. In monolayer culture the adipose derived MSCs (**A**) assumed a polymorphic, fibroblast-like morphology and could be differentiated into adipocytes (**B**; Oil red staining), osteoblasts (**C**; von Kossa) and chondrocytes (**D**; alcian blue). The isolated MSCs showed a strong positive signal for the stem cell specific markers CD90^+ ^(**E**) and CD105^+ ^(**F**) and were negative for the hematopoietic stem cell markers CD45^- ^(**G**) and CD34^- ^(**H**). Magnification: A: 5×; B: 40×; C: 20×; D: 20×; E-H: 40×.

In order to demonstrate that the cells isolated from the canine adipose tissue (Figure 1A) are indeed mesenchymal stem cells (MSCs) we differentiated them to adipocytes, osteoblasts and chondrocytes (Figure 1B, C). After three weeks' treatment with the adipogenic induction medium, the cells contained abundant amounts of vacuoles and Oil Red O staining for fat revealed that these vacuoles contained neutral lipids (B). After three weeks culture time with the osteogenic induction medium the cells changed to a more polygonal appearance, formed nodules and were stained positive with von Kossa stain for mineral deposition (C). Alcian blue staining after 14 days in culture revealed a high content of cartilage specific proteoglycans in induced cultures (D). Additionally, the isolated MSCs showed a strong positive signal for the stem cell specific markers CD90^+ ^and CD105^+ ^(Figure 1E, F). In contrast to this they were clearly labelled negative for the hematopoietic stem cell markers CD45^- ^and CD34^- ^(Figure 1G, H).

### Curcumin alone does not have a chondro-inductive effect on pure MSC high-density cultures

**Figure 2:**
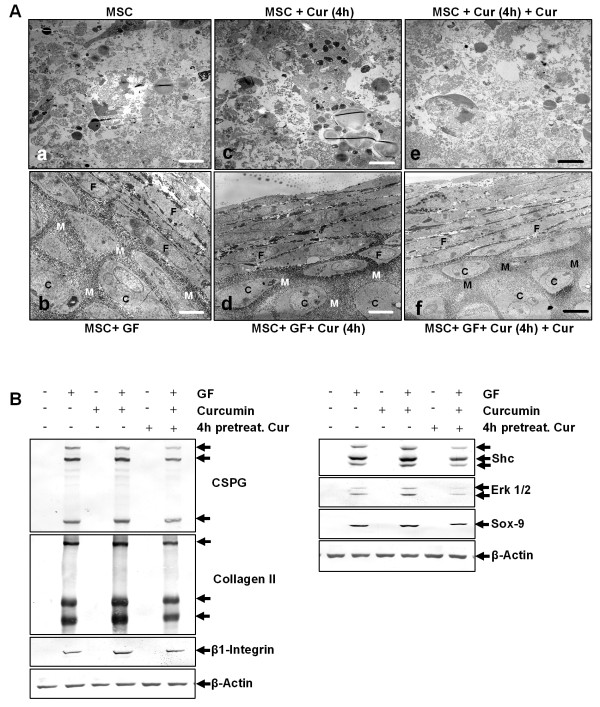
**Curcumin alone does not enhance chondrogenesis in MSCs**. **A**: 14 days high-density culture. Apoptosis or necrosis was observed in MSC cultures **(a)**, MSC cultures treated with curcumin **(c) **or MSC cultures pre-stimulated four hours with curcumin, followed by incubation with curcumin **(e)**. In contrast, chondrogenesis was observed in MSC cultures treated with the chondrogenic induction medium alone **(b)**, in combination with curcumin **(d) **or a hour-hour pre-stimulation with curcumin, followed by a combination of curcumin and the chondrogenic induction medium **(f)**. Magnification, 6000×; bar, 1 μm; C, chondrocytes; F, fibroblast-like cells; M, ECM; GF, chondrogenic induction medium. **B**: The ultrastructural findings above were confirmed by western blotting. Immunoblots of whole cell lysates were probed using antibodies that recognize CSPGs, collagen type II, β1-integrin, Shc, activated-ERK1/2 and Sox-9. Each experiment was performed in triplicate. Expression of the housekeeping gene β-actin was not affected. GF, chondrogenic induction medium.

To study the effects of curcumin on MSCs after 14 days of cultivation in three-dimensional high-density culture, ultrastructural evaluations were performed (Figure 2A). In control cultures, MSCs did not survive, but underwent apoptosis or necrosis and mainly cell debris was observed (a). Treatment of the cultures with the chondrogenic induction medium stimulated chondrogenesis (b). The formation of cartilage nodules consisting of large rounded viable cells (containing large quantities of endoplasmic reticulum, mitochondria and other cellular organelles) embedded in a fine structured, highly organised ECM was observed. Treatment of MSCs with curcumin alone did not stimulate chondrogenesis and, as in control cultures, mainly cell debris was observed (c, e). It did not make a difference whether cultures were either pre-treated for four hours with curcumin (c) or pre-treated four hours with curcumin followed by treatment with curcumin over the entire culture period (e). In contrast, in cultures treated with curcumin and the chondrogenic induction medium (d, f) chondrogenesis was observed. Chondrogenesis was similar in cultures that were either pre-treated for four hours with curcumin (d) or cultures that were pre-treated four hours with curcumin followed by treatment with curcumin over the entire culture period (f).

Western blotting was performed to confirm these results (Figure 2B). Whole cell lysates were resolved by SDS-PAGE electrophoresis and blotted onto nitrocellulose. The membranes were probed with antibodies against cartilage specific proteoglycans (CSPG), collagen type II, β1-integrins, Shc, activated extracellular regulated kinases 1 and 2 (ERK 1/2) and Sox-9.

In agreement with the ultrastructural findings, MSC cultures treated with the specific chondrogenic induction medium alone or in combination with curcumin produced high amounts of CSPGs, collagen type II and β1-integrin. Here, four-hour curcumin pre-treatment followed by treatment with the chondrogenic induction medium was as effective in inducing the production of CSPGs, collagen type II and β1-integrin as a four-hour curcumin pre-treatment followed by treatment with curcumin and the chondrogenic induction medium over the entire culture period. Further, in these cultures the chondrogenic signalling cascade was activated with high expression of Shc, activated ERK 1/2 and Sox-9. In contrast to this, cartilage specific matrix components and members of the chondrogenic signalling cascade, were not expressed in untreated cultures or cultures incubated only with curcumin. Again, it did not make a difference whether cultures were pre-treated for four hours with curcumin or pre-treated four hours with curcumin followed by treatment with curcumin over the entire culture period.

### Curcumin inhibits IL-1β activity in MSCs, enabling growth factor induced chondrogenesis

It has been reported that IL-1β inhibits chondrocyte proliferation and induces apoptosis [26,27]. We therefore evaluated the effects of curcumin on MSCs stimulated with IL-1β and/or the chondrogenic induction medium.

**Figure 3:**
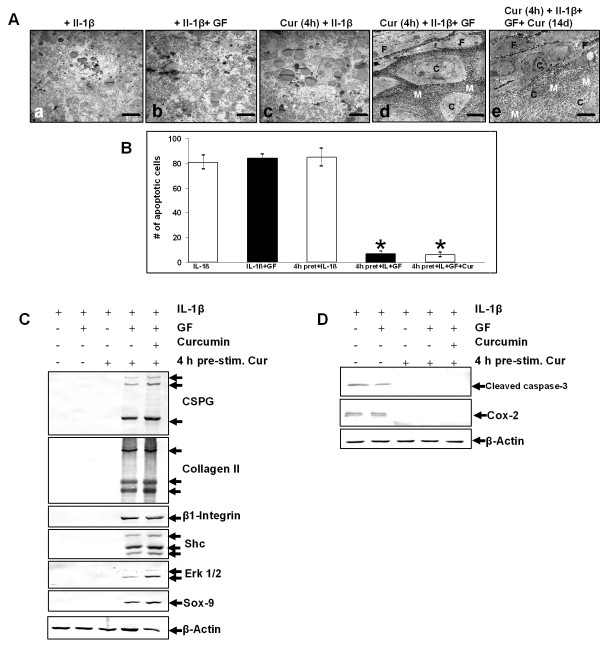
**Curcumin inhibits IL-1β activity, enabling growth factor induced chondrogenesis in MSCs**. **A**: Fourteen days high-density culture. Treatment of MSC cultures with IL-1β alone **(a)**, IL-1β and the chondrogenic induction medium **(b) **or a four-hour pre-stimulation with curcumin followed by IL-1β incubation **(c) **resulted in apoptosis or necrosis of the cells. In contrast, a four-hour pre-stimulation of MSCs with curcumin followed by incubation with IL-1β and the chondrogenic induction medium **(d) **or followed by incubation with IL-1β, the chondrogenic induction medium and curcumin **(e) **resulted in chondrogenesis. Magnification, 6,000×; bar, 1 μm; C, chondrocytes; F, fibroblast-like cells; M, ECM; GF, chondrogenic induction medium. **B**: Apoptotic cells were counted as an indicator of MSC culture degradation. In cultures stimulated with IL-1β alone, with IL-1β and the chondrogenic induction medium, or with IL-1β and curcumin the number of apoptotic cells was increased. The number of apoptotic cells remained significantly lower in MSC cultures stimulated with IL-1β, curcumin and the chondrogenic induction medium. Significant values are marked with (*). **C-D**: Immunoblots of whole cell lysates were probed using antibodies that recognize CSPGs, collagen type II, β1-integrin, Shc, activated-ERK1/2, Sox-9, activated-caspase-3 and COX-2. Each experiment was performed in triplicate. Expression of the housekeeping gene β-actin was not affected. GF, chondrogenic induction medium.

Ultrastructural evaluation revealed that stimulation of MSCs with IL-1β either alone (Figure 3A-a), in combination with the chondrogenic induction medium (b) or in combination with four-hour curcumin pre-treatment (c) resulted in apoptosis. However, treatment with IL-1β, curcumin and the chondrogenic induction medium lead to induction of chondrogenesis (d, e) with formation of cartilage nodules containing viable, rounded cells that were embedded in a cartilage specific matrix. In these cultures, a four-hour curcumin pre-treatment (d) was as effective in inhibiting IL-1β induced apoptosis as a four-hour curcumin pre-treatment followed by treatment with curcumin over the entire culture period (e).

Apoptosis was further quantified as described in Materials and Methods. The data shown in Figure 3B demonstrate a significantly increased number of apoptotic cells in cultures stimulated with IL-1β either alone, in combination with the chondrogenic induction medium or in combination with a four-hour curcumin pre-treatment. As shown at the ultrastructural level, the number of apoptotic cells significantly decreased in MSC cultures treated with IL-1β, curcumin and the chondrogenic induction medium (Figure 3B). In these cultures the number of apoptotic cells was similar between cultures that were either pre-treated for four hours with curcumin or pre-treated four hours with curcumin followed by curcumin treatment over the entire culture period.

To support these ultrastructural findings, Western blotting was performed by probing whole cell lysates with antibodies against CSPGs, collagen type II, β1-integrin, Shc, activated ERK 1/2 and Sox-9 (Figure 3C). Stimulation of MSC cultures with IL-1β either alone, in combination with the chondrogenic induction medium or with a four-hour curcumin pre-treatment did not lead to production of CSPGs, collagen type II, β1-integrin and activation of Shc, ERK 1/2 and Sox-9. In contrast, production of CSPGs, collagen type II and β1-integrin was upregulated and Shc, activated ERK 1/2 and the chondrogenic specific transcription factor Sox-9 was highly expressed in MSC cultures treated with IL-1β, curcumin and the chondrogenic induction medium. Underlining the ultrastructural findings, it did not make a difference whether cultures were either pre-treated for four hours with curcumin or pre-treated four hours with curcumin followed by curcumin treatment over the entire culture period.

Further, to demonstrate the influence of curcumin on the induction of the apoptotic signalling cascade by IL-1β in MSCs, cultures were evaluated for activated caspase-3 and the marker of inflammation and prostaglandin production COX-2 (Figure 3D). Production of activated caspase-3 and COX-2 expression was prominent in all MSC cultures stimulated with IL-1β alone and was blocked in all cultures treated with IL-1β and curcumin. Here, a four-hour pre-treatment with curcumin was as effective in inhibiting the IL-1β induced apoptotic signalling cascade as a four-hour pre-treatment with curcumin followed by curcumin treatment over the entire culture period.

### Curcumin promotes chondrogenesis in co-cultures stimulated with IL-1β

As demonstrated above, MSC cultures treated with a chondro-inductive medium undergo chondrogenesis despite the presence of IL-1β if the apoptotic and inflammatory cascades induced by IL-1β are inhibited by curcumin. Next we evaluated whether the same effect can be observed in a co-culture model of MSCs and primary chondrocytes.

**Figure 4:**
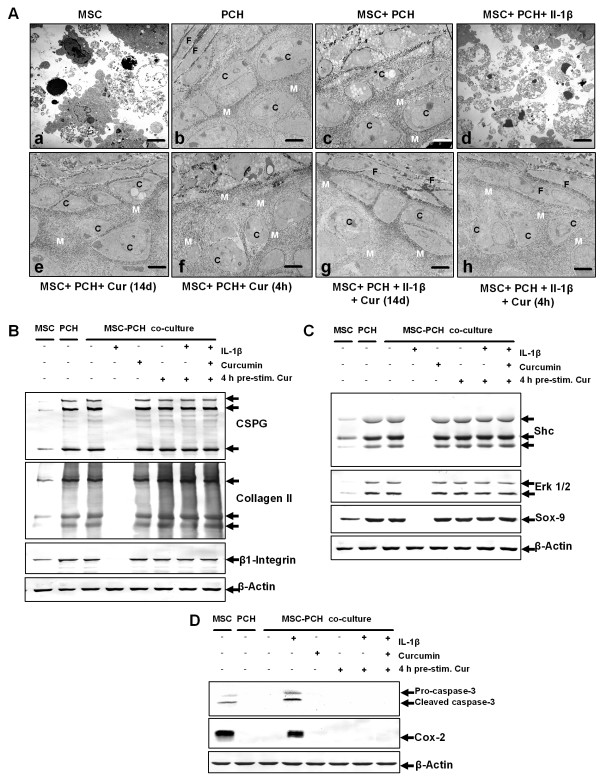
**Curcumin inhibits IL-1β activity, enabling co-culture induced chondrogenesis in MSCs**. **A**: Fourteen days high-density culture. Untreated MSC cultures became apoptotic **(a)**. In primary chondrocyte cultures **(b)**, co-cultures **(c)**, co-cultures treated with curcumin **(e) **or co-cultures pre-stimulated four hours with curcumin **(f)**, prominent chondrogenesis was observed. Stimulation of the co-culture with IL-1β alone resulted in degeneration of the cell culture **(d)**. In contrast, a four-hour pre-stimulation of the co-culture with curcumin followed by IL-1β incubation **(g) **or a four-hour pre-stimulation of the co-culture with curcumin followed by IL-1β and curcumin incubation **(h) **inhibited the adverse effects of IL-1β on the chondrogenic potential of the co-culture and prominent chondrogenesis was observed. Magnification, 6,000×; bar, 1 μm; C, chondrocytes, F, fibroblast-like cells; M, ECM. **B-D**: Immunoblots of whole cell lysates were probed with antibodies against CSPGs, collagen type II, β1-integrin, Shc, activated-ERK1/2, Sox-9, activated-caspase-3 and COX-2. In co-cultures pre-stimulated for four hours with curcumin followed either by incubation with IL-1β alone or incubation with IL-1β and curcumin, prominent production of chondrogenic matrix and adhesion molecules (B), activation of the chondrogenic signalling pathway (C) and down-regulation of apoptotic and inflammatory markers (D) was observed. Each experiment was performed in triplicate. Expression of the housekeeping gene β-actin was not affected.

Ultrastructural evaluation demonstrated cellular debris in untreated MSC cultures (Figure 4A-a), and development of cartilage nodules with rounded, viable cells embedded in a highly organised, fine structured ECM in untreated primary chondrocyte cultures (b) and in untreated co-cultures (c). Stimulation of co-cultures with IL-1β resulted in cellular degradation (d). Curcumin treatment of the co-cultures, either as a four-hour pre-treatment (e) or over the entire culture period (f) did not impede chondrogenesis. Combinational treatment of co-cultures with IL-1β and curcumin suppressed the adverse effects of IL-1β on chondrogenesis (g, h). Here, inhibition of IL-1β induced apoptosis was as effectively blocked by a four-hour curcumin treatment (g) as by curcumin treatment for the entire culture period (h).

Western blotting using antibodies against CSPGs, collagen type II, β1-integrin, Shc, activated ERK 1/2 and Sox-9 was performed to evaluate induction of chondrogenesis in the co-cultures on a molecular level (Figure 4B, C). Production of cartilage matrix specific markers and of chondrogenic signalling pathway members was slight in untreated MSC cultures and high in untreated primary chondrocyte cultures and untreated co-cultures (Figure 4B, C). Stimulation of co-cultures with IL-1β alone inhibited production and expression of CSPGs, collagen type II, β1-integrin, Shc, activated ERK 1/2 and Sox-9. In contrast, co-cultures stimulated with IL-1β and curcumin produced high levels of chondrogenic matrix specific markers (Figure 4B) and chondrogenic signalling pathway proteins (Figure 4C). Stimulation of chondrogenesis was similar between cultures that were either pre-treated for four hours with curcumin or were pre-treated four hours with curcumin followed by curcumin treatment over the entire culture period and comparable to untreated primary chondrocyte cultures and untreated co-cultures.

To further demonstrate the influence of curcumin on the induction of the apoptotic signalling cascade by IL-1β in co-cultures, cultures were evaluated for activated caspase-3 and the marker of inflammation and prostaglandin production COX-2 (Figure 4D). Activated caspase-3 and COX-2 were highly expressed in untreated MSC cultures but were not expressed in untreated primary chondrocyte cultures and untreated co-cultures. Treatment of the co-cultures with IL-1β alone led to high production of activated caspase-3 and COX-2. In contrast, in co-cultures stimulated with both IL-1β and curcumin neither activated caspase-3 nor COX-2 was detected. A four-hour pre-treatment of curcumin or curcumin treatment over the entire culture period both effectively inhibited IL-1β induced activation of caspase-3 and COX-2 production. These results confirm the ultrastructural findings described above and demonstrate that curcumin inhibits IL-1β induced apoptotic and inflammatory signalling pathways, promoting co-culture induced chondrogenesis.

### Curcumin suppresses IL-1β-induced apoptotic and inflammatory responses in MSCs in a time and concentration dependent manner

**Figure 5:**
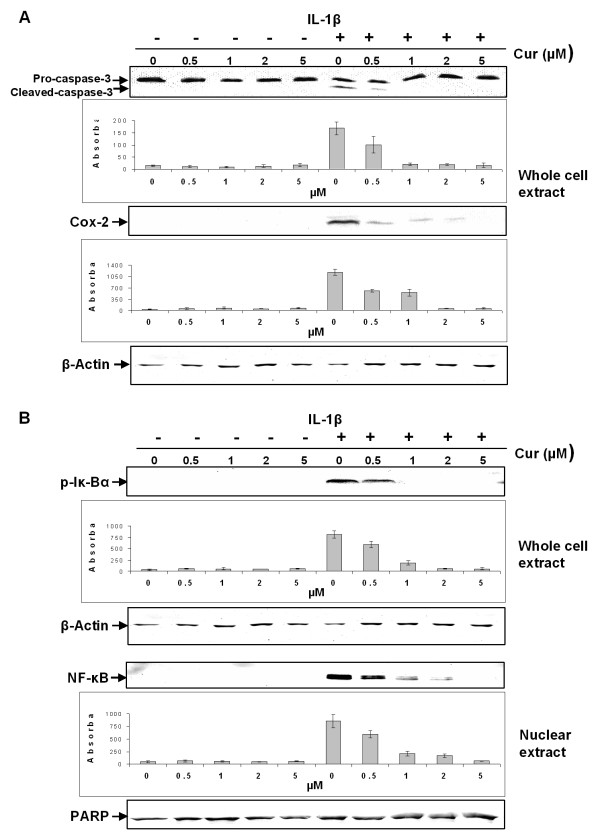
**Curcumin suppresses IL-1β-induced apoptotic and inflammatory responses in monolayers of MSCs in a *concentration dependent *manner**. Monolayer cultures of MSCs were pre-stimulated for four hours with various concentrations of curcumin (0, 0.5, 1, 2 and 5 μM) followed by 24 h incubation with IL-1β, and Western blotting was performed using whole cell lysates and nuclear extracts. **A**: A strong dose dependent effect on IL-1β induced activation of caspase-3 and COX-2 was observed. Curcumin concentrations as low as 0.5 μM suppresses IL-1β induced activation of caspase-3 and COX-2. Higher concentrations of curcumin completely inhibited IL-1β induced activation of caspase-3 and production of COX-2. This was confirmed by quantitative densitometry. The mean values and standard deviations from three independent experiments are shown. Expression of the housekeeping gene β-actin was not affected. **B**: Curcumin exerts a strong dose dependent effect on IL-1β activated Iκ-Bα in MSCs, by suppressing phosphorylation of Iκ-Bα (which is already fairly robust) and NF-κB nuclear translocation at 0.5 μM curcumin. Higher concentrations of curcumin blocked IL-1β-induced activation of Iκ-Bα and NF-κB translocation to the nucleus completely. This was confirmed by quantitative densitometry. The mean values and standard deviations from three independent experiments are shown. Expression of the housekeeping gene β-actin and the DNA repair enzyme PARP were not affected.

**Figure 6:**
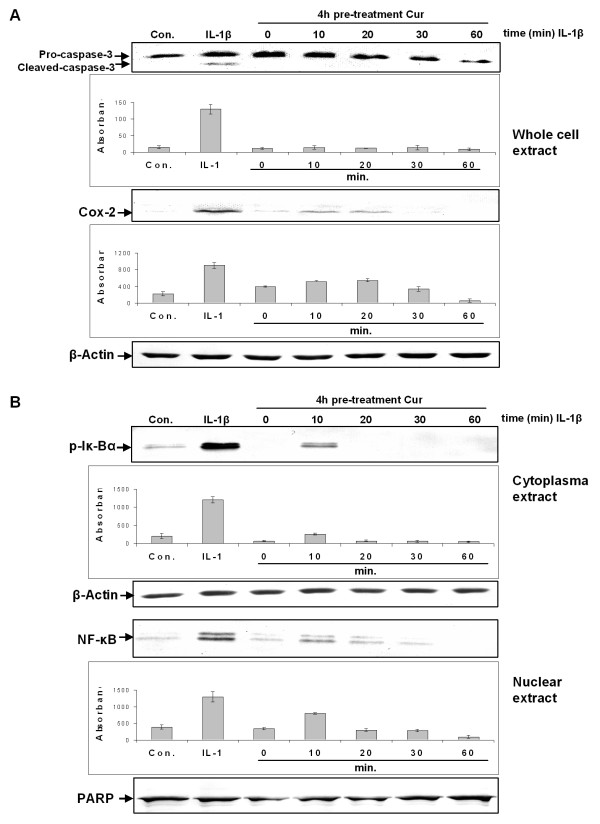
**Curcumin revokes IL-1β-induced apoptotic and inflammatory responses in monolayers of MSCs in a *time dependent *manner**. MSC Monolayer cultures were pre-stimulated for four hours with 5 μM curcumin followed by 1 h stimulation with IL-1β. Whole cell lysates, cytoplasmic and nuclear extracts were evaluated by western blotting at various time points. **A**: A four-hour pre-stimulation with 5 μM curcumin suppressed IL-1β induced activation of caspase-3 and expression of COX-2 in a time dependent manner. This was confirmed by quantitative densitometry. The mean values and standard deviations from three independent experiments are shown. Expression of the housekeeping gene β-actin was not affected. **B**: Western blotting against activated Iκ-Bα and NF-κB demonstrated that in cultures pre-stimulated with 5 μM curcumin for four hours, followed by one-hour IL-1β stimulation, neither activation of Iκ-Bα nor translocation of NF-κB to the nucleus can be demonstrated. This was confirmed by quantitative densitometry. The mean values and standard deviations from three independent experiments are shown. Expression of the housekeeping gene β-actin and the DNA repair enzyme PARP were not affected.

Further experiments were carried out to evaluate the interaction between curcumin and IL-1β in MSCs. These experiments demonstrated that curcumin suppressed IL-1β induced activation of apoptotic and inflammatory pathways in a concentration (Figure 5) and time (Figure 6) dependent manner.

As shown in Figure 5A, treatment with as little as 0.5 μM of curcumin over the entire culture period was sufficient to significantly suppress IL-1β induced activation of caspase-3 and COX-2 production in MSCs. Pro-caspase-3 production remained unaffected.

The IL-1β-activated transcription factor nuclear factor-κB (NF-κB) plays an essential role in mediating inflammatory and apoptotic processes in chondrocytes and it is known that in chondrocytes, curcumin is able to suppress NF-κB [9,14,28]. To evaluate whether curcumin influences IL-1β-induced NF-κB in MSCs, we investigated the NF-κB signalling pathway. As demonstrated in Figure 5B, curcumin suppressed IL-1β-induced activation of Iκ-Bα in MSCs. This correlated clearly with decreased NF-κB translocation to the nucleus. Inhibition of Iκ-Bα phosphorylation as well as NF-κB translocation to the nucleus was evident when curcumin was included at a concentration of 0.5 μM. Higher concentrations of curcumin completely blocked IL-1β-induced activation of Iκ-Bα and NF-κB translocation to the nucleus (Figure 5B).

Further, pre-treatment of MSC cultures with 5 μM curcumin also inhibited IL-1β-induced activation of caspase-3 and COX-2 expression in a time dependent manner (Figure 6A). After 60 minutes incubation time, activation of caspase-3 and COX-2 production was completely suppressed. Pre-treatment of MSC cultures with 5 μM curcumin also inhibited activation of Iκ-Bα and NF-κB translocation to the nucleus in a time dependent fashion (Figure 6B). In contrast, in IL-1β treated control cultures, activated caspase-3, higher expression of COX-2, activated Iκ-Bα and higher concentrations of NF-κB in the nucleus were observed.

## Discussion

The aim of this study was to evaluate whether curcumin has the capacity to modulate inflammatory processes in MSCs and thus support chondrogenesis in an *in vitro *model of OA incorporating MSCs, primary chondrocytes and pro-inflammatory cytokines.

Our observations lead to the following conclusions: (1) Curcumin itself does not have any chondro-inductive potential in MSCs, and treatment of MSCs with IL-1β leads to cell apoptosis. (2) Although co-treatment of MSCs with curcumin and IL-1β does not promote chondrogenesis, it clearly inhibits up-regulation of pro-inflammatory and apoptotic signalling cascades in MSCs; (3) If MSCs receive a chondrogenic stimulus, curcumin mediated inhibition of IL-1β-induced catabolic signalling cascades enables chondrogenic differentiation. (4) This effect is observed either by pre-treatment with curcumin (four hours) or curcumin incubation over the entire culture period. (5) Chondrogenic stimulation can be achieved either with a chondro-inductive medium or through direct, close contact co-culture of MSCs with primary chondrocytes; (6) Similar to chondrocytes, curcumin in MSCs targets the Iκ-Bα cascade, inhibiting IL-1β-induced Iκ-Bα phosphorylation and NF-κB nuclear translocation; (7) The effects of curcumin on the IL-1β signalling pathway in MSCs are time and concentration dependent.

OA and RA are characterised by high levels of pro-inflammatory cytokines in the articular joint. These are produced by synovial lining cells, macrophages and the chondrocytes themselves further exacerbating cartilage degrading and degenerative processes [29,30]. Although, as in numerous other tissues, MSC-like progenitors are also resident in adult cartilage tissue [10], these degrading and degenerative processes gradually lead to an imbalance between cartilage catabolism and anabolism, impeding MSC chondrogenesis. It has been reported that activation of NF-κB is the key to induction of inflammation during OA and RA, and that NF-κB inhibition might prove to be a potential concept for arthritis treatment [31,32].

The polyphenol curcumin, derived from the rhizomes of *Curcuma longa *is a promising therapeutic agent for the treatment of OA and RA as it has pro-apoptotic properties in synovial lining cells [33,34] and has been shown to have anti-inflammatory and anti-apoptotic effects in chondrocytes [14,15]. In chondrocytes these effects are mainly mediated by inhibiting IL-1β-induced activation of NF-κB and thus suppression of caspase-3 activation, of production of COX-2 and upregulation of MMPs [9].

In this study we demonstrate that the IL-1β-induced catabolic signalling cascade is suppressed by curcumin in MSCs as well as in chondrocytes. Further, we clearly demonstrate that IL-1β induced Iκ-Bα activation is suppressed by curcumin, resulting in suppression of NF-κB translocation to the nucleus and attenuated activation of caspase-3 and COX-2 production. Taken together, our experiments on MSCs demonstrate that time and concentration dependent effects of curcumin inhibit the induction of degradative and inflammatory pathways by IL-1β through revoking activation of caspase-3, production of COX-2, phosphorylation of Iκ-Bα and inhibition of nuclear translocation of NF-κB.

Interestingly, although we demonstrated that MSCs in high-density culture cannot survive without a chondrogenic stimulus, stimulating these untreated cultures with IL-1β and curcumin neither activated caspase-3 nor production of COX-2. This suggests that although MSCs alone in this high-density model do not survive without a necessary stimulus, they become necrotic rather than apoptotic.

We did not observe a chondrogenic effect of curcumin alone on MSCs, despite demonstrating anti-inflammatory and anti-apoptotic effects of curcumin in MSCs. However, given the necessary stimulus, chondrogenesis was observed. This demonstrates that curcumin interferes with the IL-1β induced apoptotic pathways in MSCs thus providing a suitable microenvironment allowing MSCs to undergo chondrogenesis even in the presence of IL-1β, as long as MSCs simultaneously receive the correct differentiation stimulus. This was confirmed through high production of ECM and adhesion and signalling molecules such as β1-Integrin. Similar observations have been made in chondrocytes [15]. Interestingly, integrins have already been shown in several tissues to be able to mediate curcumin action [35,36]. As β1-integrins are highly expressed in developing and adult cartilage, it is possible that the mechanism of action of curcumin in MSCs and chondrocytes might involve the β1-integrin receptor signalling pathway.

Several studies have suggested that curcumin interacts with the TGF-β signaling cascade, modulating the action of TGF-β. For example in renal cells curcumin blocks multiple sites of the TGF-β signaling [37] or Smad inhibition in human proximal tubule cells [38]. However, no previously published *in vitro *studies have explored the potential interaction between TGF-β and curcumin in chondrocytes.

In our experiments we did not observe inhibition (or a major difference in) the amounts of extracellular matrix production and signaling cascades when co-cultures were treated with the chondrogenic induction medium and curcumin or only with curcumin, we assume that here a possible interaction between curcumin and the TGF-β signaling pathway does not have an inhibitory effect on positive chondrogenic signaling. However, interaction of curcumin and TGF-β signaling in chondrocytes is an interesting point and will require further detailed investigations.

In the present study we demonstrated that a fairly low concentration of curcumin (0.5 μM) was sufficient to inhibit IL-1β induced activation of degradative pathways in MSCs. It must be noted that in this study we worked with low concentrations of curcumin, the highest concentration used being 5 μM. We chose this concentration based on previous studies in our laboratory demonstrating that canine MSCs do not tolerate curcumin concentrations higher than 5 μM. A recent study by Kim and co-workers demonstrated that chick MSCs treated with 20 μM curcumin become apoptotic and do not undergo chondrogenesis [39]. This clearly demonstrates that MSCs can only tolerate lower concentrations of curcumin in contrast to chondrocytes [40]. However, *in vivo *administered doses of curcumin in clinical trials differ greatly from this and have ranged between 2 to 10 g per day [41-43]. An explanation for these high concentrations is that intestinal absorption of curcumin is fairly low, mainly due to the fact that curcumin is practically insoluble in water, and that it has a low bio-availability [44,45]. Despite its low bio-availability, efficacy has been demonstrated in several *in vivo *studies [46-48].

In order to develop a therapeutic strategy for OA/RA treatment with curcumin it would be interesting to determine bioavailability of curcumin in the synovial fluid following various routes of administration (that is, oral, intra articular or topical). This is especially interesting as it may be also possible that *in vivo *curcumin exerts its effects via another organ (that is, the liver), which then leads to positive anabolic signalling in the joint. Therefore, to integrate our *in vitro *data with results from other studies, it is important to design and perform further *in vivo *studies. Furthermore, our experiments were carried out using canine derived cells and it may not be possible to make generalised statements about the activity of curcumin on MSC-like cells in other species.

## Conclusions

**Figure 7:**
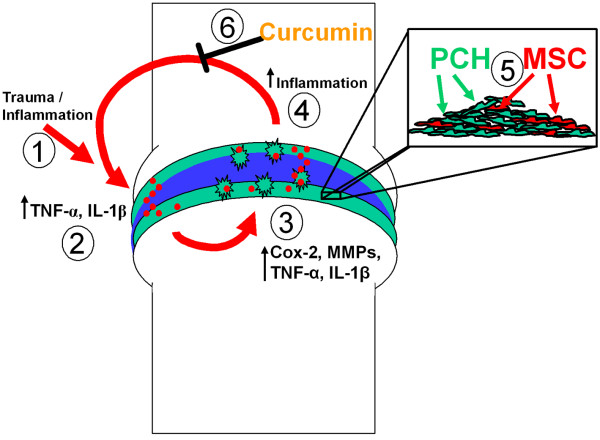
**Schematic demonstrating the inflammatory cycle that inhibits chondrogenic differentiation of MSCs in OA and the effect of curcumin**. Trauma, inflammation or a combination of both **(1) **lead to the production and accumulation of high levels of pro-inflammatory cytokines **(2) **in the joint. These trigger the production of additional pro-inflammatory cytokines which induce genes encoding prostaglandin synthesizing enzymes (that is, COX-2) and matrix degrading enzymes **(3) **such as MMPs and aggrecanases. These events promote cartilage degradation and stimulate further joint inflammation **(4) **and a self-perpetuating inflammatory and catabolic cascade develops. As illustrated, cartilage contains chondrocytes and MSC-like progenitors **(5)**. Chemical or biological agents may help create a suitable microenvironment in order for these progenitor cells to undergo chondrogenesis and regenerate new cartilage in OA. In this study we tested the hypothesis that phytochemical modulators of NF-κB can counteract this inflammatory and catabolic cascade and demonstrated that curcumin **(6) **has the capacity to block the action of pro-inflammatory cytokines in the joint thus disrupting the inflammatory cycle.

Our results suggest that curcumin, the naturally occurring NF-κB inhibitor, protects MSCs from the deleterious effects of pro-inflammatory cytokines and thus creates a suitable microenvironment for MSCs to undergo chondrogenesis and this strategy may help stimulate cartilage regeneration *in vitro *(Figure 7). We therefore propose curcumin as a potential new therapeutic for the prophylactic treatment of OA/RA and for OA/RA cases where cartilage damage is marginal.

## Abbreviations

AP303a: alkaline phosphatase linked sheep anti-mouse; AP304A: sheep anti-rabbit secondary antibodies; DMSO: dimethylsulfoxide; COX-2: cyclooxygenase-2; CSPG: cartilage specific proteoglycans; ECM: extracellular matrix; ERK 1/2: extracellular regulated kinases 1 and 2; FCS: fetal calf serum; IKK: IκB kinase; IL: interleukin; MAB1965: anti-β1-integrin antibody; MAB2015: monoclonal anti-adult cartilage-specific proteoglycan antibody; MAPK: mitogen-activated protein kinase; MMP: matrix metalloproteinase; MSC: mesenchymal stem cell; MTT: 3-(4,5-dimethylthiazol-2-yl)-2,5-diphenyltetrazolium bromide; NF: nuclear factor; NF-κB: nuclear factor-κB; OA: osteoarthritis; PAB746: polyclonal anti-collagen type II antibody; PARP: (poly(ADP-Ribose) polymerase); PBS: phosphate buffered saline; RA: rheumatoid arthritis; Shc: src homology collagen; TNF: tumor necrosis factor.

## Competing interests

The authors declare that they have no competing interests.

## Authors' contributions

CB carried out the experimental work, data collection and interpretation, and manuscript preparation. AM, UM and MS conceived of the study design and coordinated the studies, data interpretation and manuscript preparation. All authors have read and approved the final manuscript.
